# Nanomaterials against intracellular bacterial infection: from drug delivery to intrinsic biofunction

**DOI:** 10.3389/fbioe.2023.1197974

**Published:** 2023-04-27

**Authors:** Yinglu Chen, Xiaoheng He, Qiuhong Chen, Yi He, Fangman Chen, Chao Yang, Liang Wang

**Affiliations:** ^1^ Department of Orthopedics, Academy of Orthopedics-Guangdong Province, Orthopedic Hospital of Guangdong Province, Guangdong Provincial Key Laboratory of Bone and Joint Degenerative Diseases, The Third Affiliated Hospital, Southern Medical University, Guangzhou, China; ^2^ School of Biology and Biological Engineering, South China University of Technology, Guangzhou, China; ^3^ Department of Applied Chemistry, Xi’an University of Technology, Xi’an, China; ^4^ Department of Rheumatology and Immunology, The Third Affiliated Hospital, Southern Medical University, Guangzhou, China; ^5^ State Key Laboratory of Quality Research in Chinese Medicine, Institute of Chinese Medical Sciences, University of Macau, Macau, Macau SAR, China

**Keywords:** intracellular infection, drug delivery, antibacterial, reactive oxygen species, bioactive nanomaterials

## Abstract

Fighting intracellular bacteria with strong antibiotics evading remains a long-standing challenge. Responding to and regulating the infectious microenvironment is crucial for treating intracellular infections. Sophisticated nanomaterials with unique physicochemical properties exhibit great potential for precise drug delivery towards infection sites, along with modulating infectious microenvironment via their instinct bioactivity. In this review, we first identify the key characters and therapeutic targets of intracellular infection microenvironment. Next, we illustrate how the nanomaterials physicochemical properties, such as size, charge, shape and functionalization affect the interaction between nanomaterials, cells and bacteria. We also introduce the recent progress of nanomaterial-based targeted delivery and controlled release of antibiotics in intracellular infection microenvironment. Notably, we highlight the nanomaterials with unique intrinsic properties, such as metal toxicity and enzyme-like activity for the treatment of intracellular bacteria. Finally, we discuss the opportunities and challenges of bioactive nanomaterials in addressing intracellular infections.

## 1 Introduction

In the territory of infectious disease, chronic and persistent infections caused by intracellular bacteria pose a thorny threat to public health (Kamaruzzaman et al., 2017). In these contexts, pathogens such as *Staphylococcus aureus* (*S. aureus*), *Mycobacterium tuberculosis* (*M. tuberculosis*), *Salmonella* and *Listeria* are able to nestle in professional phagocytic cells, particularly macrophages, which not only shield them from the host immune system’s eradication but also from antibacterial agents. The most prominent intracellular infections in clinic are associated with *M. tuberculosis*, which can require prolonged and substantial antibiotic treatments. Over extended periods, intracellular bacteria can act as a ‘Trojan horse’, resulting in a secondary relapsing infection primarily due to their ability to survive and multiply rapidly within host cells. This category includes obligate intracellular bacteria that can reproduce both inside and outside their cellular hosts, as well as facultative intracellular bacteria that depend on host cells for their reproduction (Briones et al., 2008).

To date, various families of antibiotics, such as rifampin, isoniazid, and linezolid, are commonly used in clinical settings to treat intracellular bacterial infections. However, these antibiotics are often ineffective in completely eradicating intracellular pathogens. This difficulty in treating intracellular infections is largely due to two factors: the inability of sufficient antibiotics to penetrate infected cells, and the various mechanisms by which bacteria can escape host cells. On the one hand, many antibiotics are hydrophilic and have poor intracellular permeability, which limits their effectiveness in treating intracellular infections. On the other hand, even antibiotics are able to diffuse into cells, they may be inactivated by several factors within cells, such as degradation by acidic, redox, or multi-enzymatic microenvironment, or discharge by efflux pumps (Wright, 2005). Accordingly, the restricted cellular penetration and intracellular instability of antibiotics cause sub-therapeutic concentrations within cells, resulting in the failure of anti-intracellular bacteria and the long-lasting persistence of pathogens.

Phagocytic systems are not only capable of killing invading pathogens, but also acting as a natural shield in some cases, preventing bacteria from being eliminated by antibiotics. However, intracellular bacteria develop some mechanisms to evade the innate immune response. These mechanisms include escaping from endosomal/lysosomal/phagolysosomal compartments to the cytoplasm, preventing the fusion of phagosomes and lysosomes, or developing resistance to the bactericidal microenvironments found in lysosomes/phagolysosomes. For instance, *Salmonella enterica* is usually located in late endosomes (Brouillette et al., 2003), while *Mycobacterium tuberculosis*, the typical intracellular bacterium, survives within phagosomes (Peng et al., 2016). This leads to those antibiotics that can penetrate the cell membranes are unable to eliminate evasive *M. tuberculosis* due to their failure to concentrate in phagosomes (Onyeji et al., 1994). Taken together, bacteria with different escape mechanisms survive in distinct cell compartments, making it difficult for antibiotics to locate within the appropriate compartments and resulting in inactive antibacterial effects.

Compared with the deficiency of traditional antibiotics against intracellular bacteria, targeted drug delivery systems show promising potentials for the management of intracellular infections through improving the cellular uptake and distribution of antibiotics. With the advances in nanotechnology, a wide variety of nanomaterials have been designed for controlled delivery of anti-bacterial agents, achieving maximal therapeutic efficiency along with minimizing potential adverse effects. Furthermore, the unique intracellular microenvironment at infected sites, with a low pH, redox potential, abundant H_2_O_2_, bacterial and cellular enzymes, can serve as responsive stimulus to realize spatiotemporal drug release of nanomaterials. Thus, exploring sophisticated nanomaterials with unique bioactivities in response to the factors or cues of intracellular microenvironment offers great potentials in eliciting specific responses and functions to kill intracellular bacteria. In these contexts, a deep understanding of the interactions between cells, intracellular bacteria and nanomaterials may provide guidance to develop intelligent nanomaterials with precise environmental responsiveness for efficient and safe management of intracellular infections.

**FIGURE 1:**
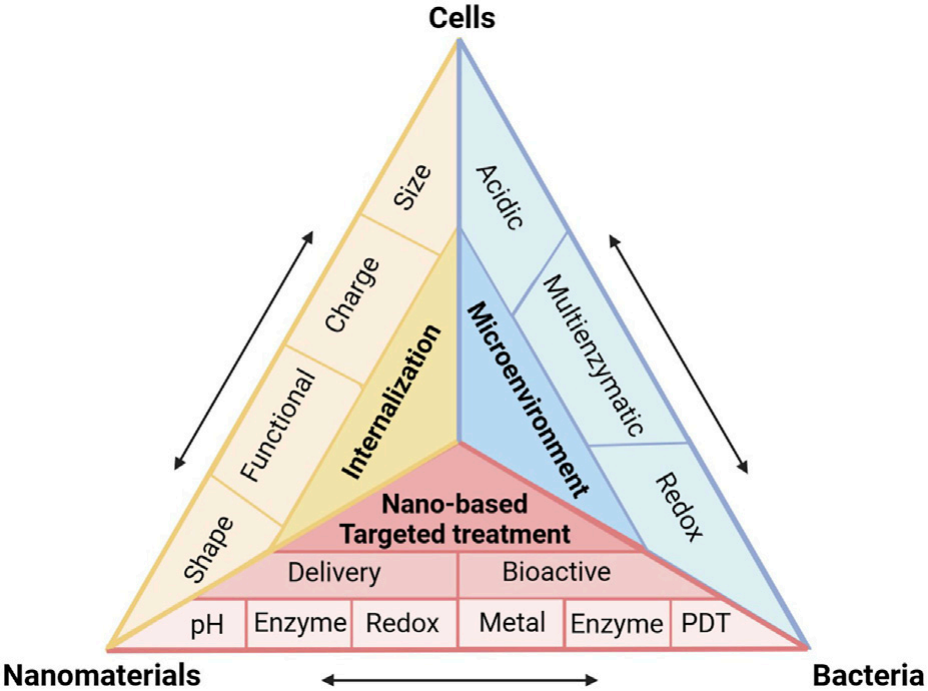
Schematic of interactions between cells, intracellular bacteria and nanomaterials during employing multifunctional nanomaterials against intracellular infections.

In this review, we identify the key characters and therapeutic targets of intracellular infection microenvironment. We illustrate how the nanomaterials physicochemical properties, such as size, charge, shape and surface functionalization affect the interaction between nanomaterials, cells and bacteria. We introduce the recent progress of nanomaterial-based targeted delivery and controlled release of antibiotics in intracellular infection microenvironment. We highlight the nanomaterials with unique intrinsic properties, such as metal toxicity and enzyme-like activity for the treatment of intracellular bacteria (Figure 1). We discuss the opportunities and challenges of bioactive nanomaterials in addressing intracellular infections.

## 2 Intracellular bacteria and microenvironment

### 2.1 Host bactericidal mechanisms

When pathogens invade, macrophages, as one of the major immune cells, fight against infection by expressing a series of receptors to trigger innate immunity. This surveillance recognition is achieved through sensors called pattern recognition receptors (PRRs) that detect pathogen-associated molecular patterns (PAMPs) and damage-related molecular patterns (DAMPs) (Plüddemann et al., 2011; Broz and Monack, 2013). After the detection of pathogens, phagocytosis plays an essential role in anti-bacterial host defense, which is mainly manifested by the effective internalization of pathogens. In such a conversion, the engulfment of pathogens by macrophages and a series of sequent membrane remodeling leads to the formation of a membrane-bound vesicle named phagosome. Following the internalization, it is a clearance process in which the phagosome acquires bactericidal and degradative functions termed phagosomal maturation. At the terminal stage of its maturation, the microenvironment of the phagosome becomes highly acidic, degradative, and oxidative, which all contribute to clearing the invaded pathogens. The low pH in phagosomes is related to the progressive acidification within the vacuole, which is realized by the V-ATPase-mediated proton pump (Flannagan et al., 2009). Meanwhile, this acidic condition favors the subsequent formation of phagolysosomes and optimal enzymatic activity of hydrolases in lysosomes.

Another mechanism to kill bacteria is the delivery of molecules with degradative functions, such as defensins, cathelicidins, lysozyme and hydrolases, into phagosomes. Defensins are able to permeabilize bacteria membranes due to the formation of ion transport channels. Cathelicidins, on the other hand, induce permeabilization acting on the cell wall as well as both the outer and inner membranes of bacteria. In addition, hydrolases targeting carbohydrates and lipids also exist in phagosomes, which degrade a wide range of invading bacterial components (Flannagan et al., 2009).

Phagocytes could also act through a large amount of reactive oxygen species (ROS) and reactive nitrogen species (RNS) produced by the NOX2 NADPH oxidase (Quinn and Gauss, 2004; Minakami and Sumimoto, 2006) and NOS2 nitric oxide synthase (Fang, 2004). The transfer of electrons from NADPH to molecular oxygen results in the formation of superoxide radicals (O_2_·^−^), which are released into phagosomes (Quinn and Gauss, 2004; Mizushima et al., 2011). After that, O_2_·^−^ in phagosomes react with H_2_O_2_ to produce hydroxyl radicals (·OH) and singlet oxygen (^1^O_2_) (Minakami and Sumimoto, 2006). It is worth noting that the fusion of the lysosomes and vacuoles leads to partial disruption of the membrane connection, releasing the contents, such as myeloperoxidase, into the phagosomes. Myeloperoxidase within the phagosomes can catalyze abundant H_2_O_2_ and halogen ions to highly bactericide hypochlorous acid (HClO) and chloramines (Thomas, 1979; Shepherd, 1986). RNS is also essential antimicrobial effectors, which reacts with ROS to destroy pathogens, causing nitrosative stress. The production of RNS starts with NOS catalyzing *L*-arginine and citrulline to produce nitric oxide (NO), which begins with superoxide to form peroxynitrite (ONOO^−^), which is a highly reactive species that can directly act with several biological targets and cell components, including lipids, amino acid residues, and DNA bases (Webb et al., 2001). Besides, peroxynitrite also has the ability to get across cell membranes to some subcompartments like phagosomes through anion channel (Fang, 2004).

Accordingly, those produced free radicals and oxidation-state components contribute to protein denaturation and lipid peroxidation by oxidative damage, which leads to irreversible damage to the invading bacteria (Boyle and Randow, 2013). This natural defense process provides ideas for biomimetic strategies to eliminate intracellular bacteria.

### 2.2 Bacterial defensive mechanisms

Unlike extracellular bacteria, intracellular bacteria that can survive and replicate in host cells, especially macrophages, adapt to challenging intracellular microenvironment and evolve intelligent mechanisms to evade host clearance. Those intracellular bacteria are typically divided into two categories: phagosomal and cytosolic bacteria. Most intracellular bacteria studied to date are stored in phagosomes. In detail, the phagosomes provide a safe haven for bacteria, shielding them from immune system detection, and facilitating their replication using the components within the phagosomes (Cullinane et al., 2008; Lamkanfi and Dixit, 2010). Alternatively, cytosolic bacteria benefit from rich nutrient conditions and a relatively spacious microenvironment, enabling them to survive in the host cell cytoplasm despite the presence of immune defenses. In general, treatments that can eliminate bacteria in phagosomes can also act on cytosolic bacteria. It is much thornier to treat phagosome bacteria than cytosolic ones, so our review focus on phagosome bacteria.

Survival of intracellular bacteria presents three main challenges: evading immune system surveillance, resisting the microenvironment, and evading the phagolysosomal pathway. Numerous strategies, including actin-based cell-to-cell spread, low expression of flagellin, avoidance, blockage and adaptation to the phagolysosomal pathway, are utilized by intracellular pathogens.

Different species of bacteria have their own intracellular lifestyles. The majority of phagosome bacteria have abilities to prevent phagosomes maturation, the terminal stage before lysosomes fusion (Ray et al., 2009). For instance, some bacteria have evolved metabolic pathways to prevent acidification in phagosomes, or express specific proteins to withstand low pH microenvironment (Park et al., 1996; Vandal et al., 2008; Huang et al., 2009; Martinez et al., 2011). In addition, some bacteria are able to express detoxifying enzymes (Schmidtchen et al., 2002) like catalase or superoxide dismutase to balance ROS/RNS levels within phagosomes (John et al., 2001; Ng et al., 2004), or interfere with the biological function of enzymes that catalyze ROS/RNS production (Mott et al., 2002). As a result, bacteria protect themselves from being destroyed and eliminated by ROS/RNS (Rudel et al., 2010; Ashida et al., 2011).

Other bacteria escape into the cytoplasm, which constitutes a wild and favorable microenvironment, through sophisticated mechanisms, such as escaping from vesicles, and permeabilizing phagosomes. For those bacteria, it is essential to escape the phagosome subcompartments as early as possible after internalization to avoid fusion with lysosomes. In this process, protein secretion plays a key role in allowing bacteria to cross the cytoplasmic membranes, cell walls, vacuole and host cell membranes, to achieve both intracellular vacuole spread and cell-to-cell spread. Among them, the four major protein secretion ssystems are the type III secretion system (T3SS) (Cornelis, 2006), type IV secretion systems (T4SSs) (Vogel et al., 1998; Christie and Cascales, 2005), type VI secretion systems (T6SSs) (Coulthurst, 2013; Kudryashev et al., 2015) and type VII secretion system (T7SS) (Abdallah et al., 2007), which hold the potential to serve as inhibitory targets for treatment design.

With these findings in mind, a comprehensive understanding of the mechanisms by which intracellular pathogens evade host cells and respond to the innate immune systems are critical to elucidate the pathogenesis of intracellular infections. Moreover, systematically deciphering the interactions between host cells and intracellular pathogens may provide clues for designing advanced nanotherapeutics against intracellular infections.

## 3 Interactions between bacteria, cells, and nanomaterials

As discussed above, most intracellular bacteria spend their whole lifestyles within phagosomes. Internalization and phagosomal maturation are essential to keep phagocytosis effective. After being detected and taken up by phagocytes, bacteria undergo a series of membrane fusions and interactions, resulting in their entrapments in sub-compartments such as phagosomes or vacuoles. However, since bacteria may be located in different phagosomes, tailored designed nanomaterials against intracellular pathogens require coexisting in the same compartments. Therefore, a deep understanding of the interactions among bacteria, phagocyte cells, and nanomaterials is essential for the principle of material design.

Different nanomaterials may be located in different sites relative to cells, such as the inner or outer cell membranes, and can influence cell proliferation, apoptosis, and migration. Therefore, understanding the relevant parameters in nanomaterials interactions with cell membranes is essential in regulating nanomaterials internalization. Nanomaterials can be internalized into cells via phagocytosis, diffusion, and fluid phase endocytosis (He et al., 2009). The endocytosis refers to the process by which the plasma membrane invaginates and forms vesicles, thereby transporting extracellular compositions into cells. It is categorized into four major types, including clathrin/caveolae-dependent endocytosis, phagocytosis, pinocytosis, and macropinocytosis (Doherty and McMahon, 2009; Howes et al., 2010; Sahay et al., 2010; Sandvig et al., 2011). Most nanoparticles enter cells through endocytosis, while relatively few enter via other mechanisms. Several factors, including size, shape, surface charge and functionalization, have influence on the uptake of materials by cells.

### 3.1 Size-dependent cellular uptake

Unlike non-phagocytes, which tend to engulf spherical nanoparticles in the 20–50 nm range, phagocytes preferentially take up micro scale particles (González et al., 1996; Champion et al., 2008; Jiang et al., 2008). Experimental results for silver nanoparticles have shown that well-dispersed 20–200 nm particles were internalized better by non-phagocytes than phagocytes (Lankoff et al., 2012), whereas aggregated silver particles were more likely to be internalized by phagocytes (Wang et al., 2012). The same phenomenon has been found for smaller nanoparticles like iron oxide particles, where small iron oxide particles exhibited a higher level of phagocytic accumulation than ultra-small particle size particles (Raynal et al., 2004).

This difference in internalization may be attributed to the influence of size on internalization pathways. Normally ultra-small particles are not recognized as exogenous agents by macrophages and can enter directly into cells based on pores in the cell membranes, whereas microscale particles are more likely to be absorbed by the reticuloendothelial system. Besides, smaller sizes possess larger surface areas, which contributes to particles diffuse into cells. When the diameter of the nanospheres is less than 200 nm, their penetration is mainly regulated by the clathrin pathway, but when the size increases to 500 nm, their internalization is mainly mediated by caveolae pathway (Rejman et al., 2004). The effect of size is also significant in the uptake of different sized anionic polystyrene particles, with smaller particles being taken up mainly through clathrin-independent cavelae-independent pathways, while larger particles are uptaken via clathrin-mediated endocytosis (Lai et al., 2007). This distinction can be explained by the fact that the clathrin-mediated pathway has a higher uptake rate than clathrin-independent cavelae-independent pathways, resulting in particles internalized through this pathway exhibiting faster accumulation within cells.

### 3.2 Charge-dependent cellular uptake

Surface charges also play a key role in cellular uptake. It is mainly manifested in the fact that the neutral surface charge nanomaterials have a lower plasma protein adsorption rate, accompanied by a longer blood circulation time, which results in a higher cell uptake due to a longer margin from the phagocytes.

Positively charged nanomaterials bind to negatively charged cell membranes through electrostatic interactions (Tahara et al., 2009; Duceppe and Tabrizian, 2010). A mass of works has demonstrated that positively charged particles were internalized into cells at a higher degree than their respective anionic particles, such as gold and silver particles, iron oxide particles, silicon dioxide, chitosan, liposome, and polymers. Besides, for negatively charged nanomaterials, the internalization decreases with increasing surface charge, while positively ones, on the contrary, performs a positive correlation with a certain range of surface charge. Surprisingly, a representative example is antibacterial silver nanoparticles coated with chitosan (CS-AgNPs) with enhanced antibacterial effect (Jena et al., 2012). The designed CS-AgNPs exhibited a significantly improved therapeutic effect on intracellular bacteria mainly due to their strong cell internalization.

Several other researches have shown some contradictory results, which might contribute to negatively charged nanoparticles can promote cellular uptake via regulating the formation of aggregation and cluster after initial electrostatic repulsion. Taking the uptake of liposome with different surface charges as an example, a negatively charged PLGA-lipid hybrid system performed better uptake behavior compared to a positively charged system (Maghrebi et al., 2020). Another hypothesis is that bacterial surfaces also present a negative charge, and phagocytes may preferentially take up anionic particles (Fröhlich, 2012). Moreover, some surface groups with negative charges, such as citrate groups, can improve the stability of nanoparticles in culture media and increase their affinity for cell membranes (Kolosnjaj-Tabi et al., 2013).

### 3.3 Shape-dependent cellular uptake

Shape is another important factor influencing the internalization of nanomaterials, especially when endocytosis pathways of nanomaterials need to be mediated by receptors. Although the surface area of rod-shaped nanomaterials is smaller than that of spherical particles, the limited binding sites on its surface can more efficiently recognize and bind to target cell surface receptors due to its aspect ratio. This allows rod-shaped nanomaterials to exhibit higher cell adhesion efficiency than spherical ones (Kolosnjaj-Tabi et al., 2013; Shao et al., 2017). On the other hand, nanomaterials with sharp shapes such as spines are able to locate in the cytoplasm due to their better ability to penetrate membranes, which enables them to remain in cells benefiting from low exocytosis (Chu et al., 2014).

### 3.4 Surface functionalization-dependent cellular uptake

**FIGURE 2:**
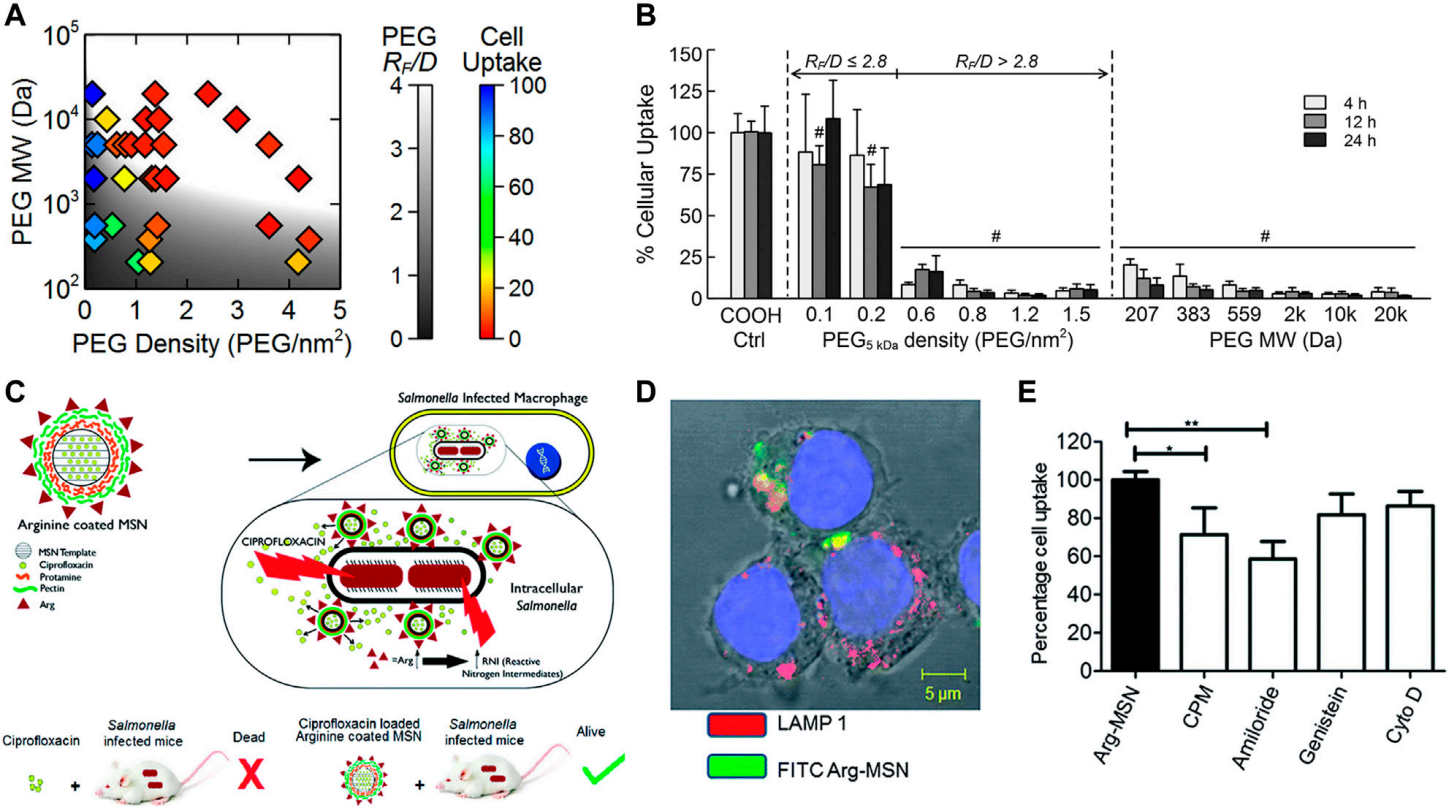
**(A,B)** Internalization probability of PEG functionalized nanoparticles with various grafting densities by differentiated human THP-1 cells. **(C)** Schematic illustration of the surface arginine-modified MSN co-localized with intracellular *Salmonella*, resulting in efficient antibiotic delivery. **(D)** Cellular trafficking and mechanism of endocytosis of Arg-MSN in RAW 264.7 cells. **(E)** Cellular uptake of Arg-MSN particles in the presence of pharmacological inhibitors of endocytosis. Reproduced with permission from Yang et al. (2014) (Copyright 2014 American Chemical Society) and Mudakavi et al. (2017).

Surface functionalization of nanomaterials not only regulates surface charge, but also improves properties such as hydrophobicity and softness. The most common strategy is surface polyethylene glycolation (PEGylation), which is mainly used to reduce the hydrophobicity of nanomaterials and alter their biological characteristics. PEGylation forms a hydrated layer that reduces the adsorption of serum proteins, increases the hydrophilicity, and reduces macrophage uptake, which is often referred to the “stealth effect” (Figures 2A, B) (Yang et al., 2014; Sanchez et al., 2017). Additionally, the difference in uptake may also be related to the change in particle softness by functionalized modification, where softer particles are more likely to be taken up by macrophages. Surface functionalization also enables active targeting of nanomaterials. For example, nanoparticles modified with polysaccharides could interact with specific receptors on cell membranes, resulting in active targeting for more precise internalization. Surface arginine-modified mesoporous silica nanoparticle (MSN) was able to co-localize with intracellular *Salmonella*, leading to efficient antibiotic delivery (Figures 2C–E) (Mudakavi et al., 2017).

## 4 Nanoparticulate materials against intracellular bacteria

Due to the particularity of intracellular bacteria, three subjects, cells, bacteria and nanomaterials included, should be taken into consideration at the same time for nano-therapy design. For instance, positively charged materials have a higher likelihood of being taken up by cells and can also bind to negatively charged bacterial surfaces through electrostatic interactions. Yang et al. utilized this strategy by designing surface cation-targeted peptide-modified MSN to deliver gentamicin, and the results proved the ability of nanoparticles to specifically target *S. aureus* and internalize into RAW 264.7 cells, indicating potentials for fighting against intracellular infections (Yang et al., 2018). Similarly, Maya et al. developed *o*-carboxymethyl-coated chitosan to achieve tetracycline targeted delivery to the infectious sites of intracellular *S. aureus* (Maya et al., 2012).

Currently, the strategies of targeting infected cells involve non-specific electrostatic interactions and specific receptor-ligand interactions, the most representative of which are mannose receptor, CD44 receptor, tuftsin receptor, and hyaluronic acid ligand. On the other hand, direct targeting of bacteria is mainly achieved through non-specific electrostatic interactions, ligand-receptor recognition, and antigen-antibody specific binding.

### 4.1 Nanocarriers

According to the above discussion, bacteria invade cells and locate in different sub-compartments, making it crucial for nanotherapeutics to deliver antibiotics in on-demand manner. This approach can improve treatment efficacy and reduce toxicity to normal tissues. Furthermore, bacterial invasion creates a unique infection microenvironment characterized by low pH, related enzyme secretion, and slight temperature changes. These conditions can be leveraged as stimulus to achieve drug targeting and release.

#### 4.1.1 pH-responsive release

**FIGURE 3:**
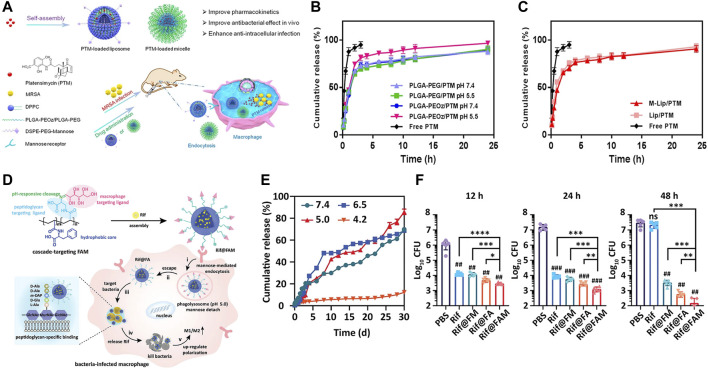
**(A)** Schematic illustration of PTM-loaded liposomes and micelles with pH-responsive release demonstrating a notably high antibacterial efficacy. **(B,C)** The pH-dependent release characteristics. **(D)** Schematic illustration of pH-triggered cascade release of rifampicin from DDS to eliminate intracellular MRSA. **(E)** The pH-sensitive release profile of DDS. **(F)** The intracellular bactericidal effect of different treatments. Reproduced with permission from Wang et al. (2020) (Copyright 2020 American Chemical Society) and Feng et al. (2022).

Progressive acidification of phagosomes and the acidic microenvironment of lysosomes are the most common stimulus for antibiotic release (Ninan et al., 2016; Pei et al., 2017; Pei et al., 2017; Lu et al., 2018; Qu et al., 2018; Su et al., 2018; Mohebali and Abdouss, 2020; Wang et al., 2020). Platensimycin (PTM), a promising natural product drug, was designed to be loaded in pH responsive release polymers, which demonstrate significantly reduce residual methicillin-resistant *Staphylococcus aureus* (MRSA) in macrophage cells (Figures 3A–C). Antituberculosis drug-loaded MSNs equipped with a pH-sensitive valve (β-cyclodextrin) were constructed to optimize loading and achieve specific intracellular delivery of drug for the treatment of tuberculosis (TB) (Clemens et al., 2012). Greater therapeutic efficacy was achieved which may be attributed to the fact that it can only be released in acidified phagosomal conditions. Other drug delivery systems (DDS) were synthesized to realize cascade release of rifampicin after Schiff base cleavage in acidic phagolysosome (Figures 3D–F) (Feng et al., 2022). This DDS in a cascade manner performed outstanding targeting and killing activity against MRSA inside macrophages. In addition, some nanomaterials with tailored surface charge have been prepared. This particular type of nanoparticles typically perform positive surface charges only under acidic conditions, which allows them to better cross the cell membranes and bind to bacteria with negatively charged surfaces. In such a scenario, nanomaterials not only exhibit efficient internalization, but also have lower toxicity to normal cells. This is because cationic particles are more likely to cause hemolysis and cytotoxicity, which can lead to lysosomal and mitochondrial damage and cell membranes destruction.

#### 4.1.2 Enzymes-responsive release

**FIGURE 4:**
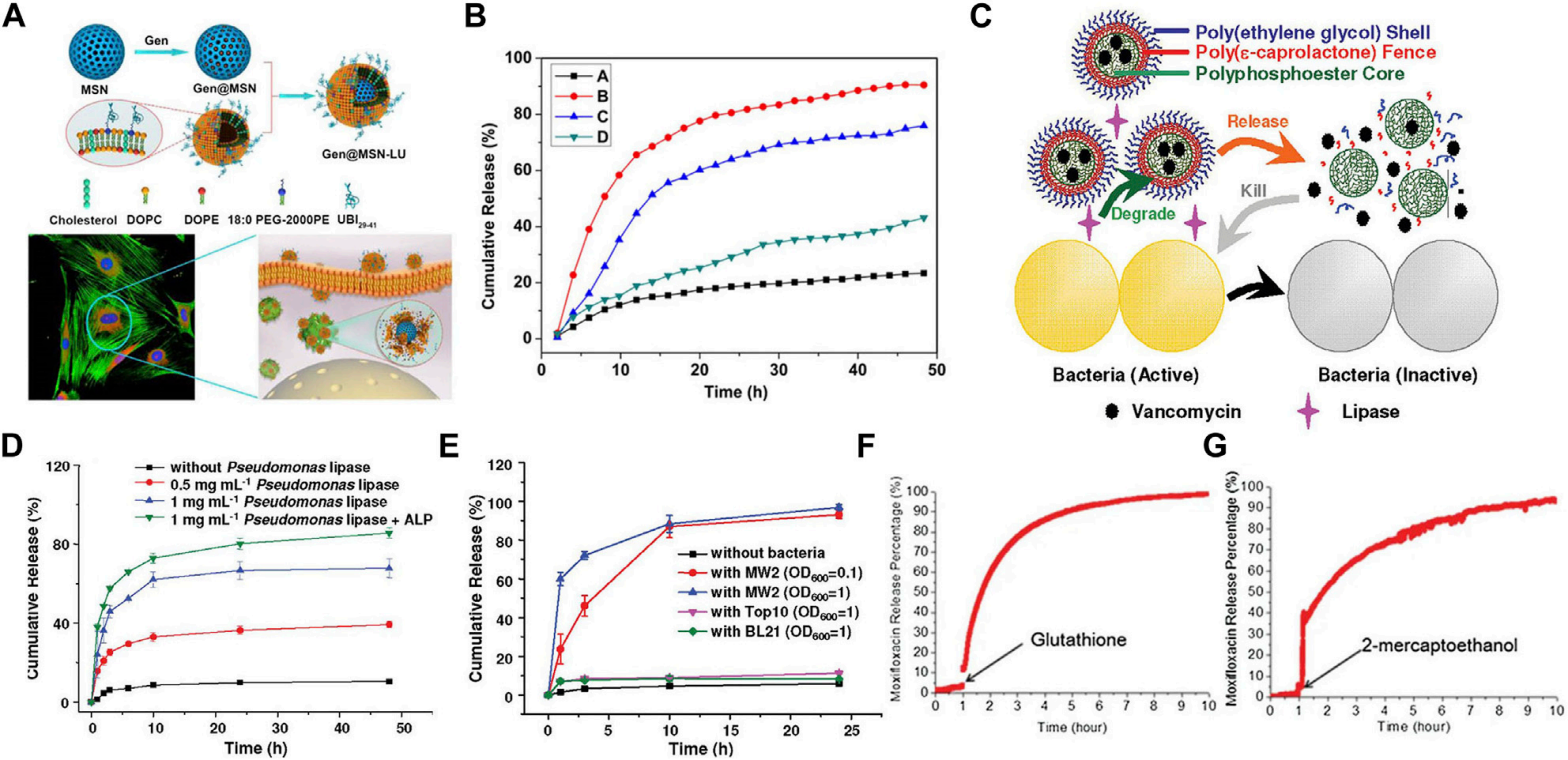
**(A)** Schematics of Gen@MSN-LU, in which the outer layer of liposomes can be degraded by the bacterium-secreted toxins, resulting in the Gen release. **(B)** The release profile of Gen from Gen@MSN-LU in different treatments (A control; B in the presence of *S. aureus*; C with lipase; D with lipase inhibitors in the presence of *S. aureus*). **(C)** Schematics of bacterial lipase triggered release of vancomycin to treat the bacterial infections. **(D,E)** Cumulative release of vancomycin from vancomycin-loaded TLN in different treatments. **(F,G)** The behavior of the redox-triggered release of Moxifloxacin. Reproduced with permission from Yang et al. (2018) (Copyright 2018 American Chemical Society), Xiong et al. (2012a) (Copyright 2012 American Chemical Society), and Lee et al. (2016).

As mentioned earlier, during the process of phagosomes maturation, various enzymes like lipase (Jaeger et al., 1994; Jaeger and Reetz, 1998), phosphatase or phospholipase (DeVinney et al., 2000) are secreted and recruited into phagosomes. Accordingly, these cellular enzymes (Alkekhia et al., 2022), bacterial enzymes (Sunnapu et al., 2022) and bacterium-secreted toxins (Figures 4A, B) (Yang et al., 2018) can also be used as stimulators for corresponding release. Peng et al. (2020) provided a practicable strategy to realize intracellular rapid release of antibiotics triggered by both enzymes and acid, thus promoting efficiency against intracellular infections. A rationally designed polymer, which was able to be degraded by bacterial lipase, significantly increased intracellular concentration of ciprofloxacin, thereby enhancing its therapeutic efficacy.

In addition, some nanogels were designed to respond to enzymes (Chen et al., 2020). Xiong et al. prepared two different feasible nanogels, a triple-layered polymer nanogel (Figures 4C–E) (Xiong et al., 2012a) and a core-shell mannose-modified nanogel (Xiong et al., 2012b), to inhibit the growth of intracellular *S. aureus*. In two systems, bacteria-secreting lipase and phosphatase or phospholipase were acted as triggers to release antibiotics, respectively.

#### 4.1.3 Redox-responsive release

Nanomaterials that can respond to the redox environment could be a potential therapeutic strategy for the treatment of intracellular infections. A functionalized MSN was used to selectively release drugs intracellularly in response to the GSH/GSSG species (reduced glutathione/oxidized glutathione disulfide), which were commonly found intracellularly (Figures 4F, G) (Lee et al., 2016). The reducing microenvironment can also be utilized to break the disulfide bonds of red blood cell (RBC) nanogels, resulting in the rapid release of antibiotics (Zhang et al., 2017).

### 4.2 Bioactive nanomaterials

Beyond the application as drug delivery carriers, some nanomaterials with intrinsic antimicrobial bioactivities have recently shown promising aspects for potential clinical applications against intracellular bacteria.

#### 4.2.1 Metal ion-based nanobactericides

Given that metals are traditionally used as drug delivery carriers, recent studies have also shown that some biomaterials with intrinsic antimicrobial bioactivity hold promise against intracellular infections. For example, gold nanomaterials can reduce the attachment of tRNA to ribosome units, decrease membrane viability and cause bacterial death. Copper ions can inhibit bacterial DNA replication and induce bacterial death through ROS production and lipid peroxidation. The release of metal ions, such as Ag^+^, Cu^2+^, and Fe^2+^, also can enhance the antibacterial effect. For instance, ZnO has the ability to inhibit the formation of biofilms and catalyze the production of ROS. Besides, the release of Zn^2+^ can also increase the permeability and degradability of membranes.

The most widely used metal for antibacterial activity is silver, which exerts its effects through a variety of mechanisms, such as lipid peroxidation, ROS generation, interference with cell wall synthesis, and increasing membrane permeability. Besides, Ag^+^ hydrolyzes bacterial macromolecules by driving the generation of hydroxyl radicals. Aurore et al. (2018) designed Ag-NPs with synergistic antimicrobial effects against *S. aureus* in human osteoclasts. The promoted bactericidal activity not only came from the direct toxicity of silver itself but also from the ROS production in osteoclasts induced by Ag-NPs. Taken together, these surprising results indicate that silver nanoparticles can be used as an effective treatment for chronic long-term infections caused by intracellular bacteria, where conventional antibiotics are difficult to achieve equivalent therapeutic effects to their extracellular effects.

**FIGURE 5:**
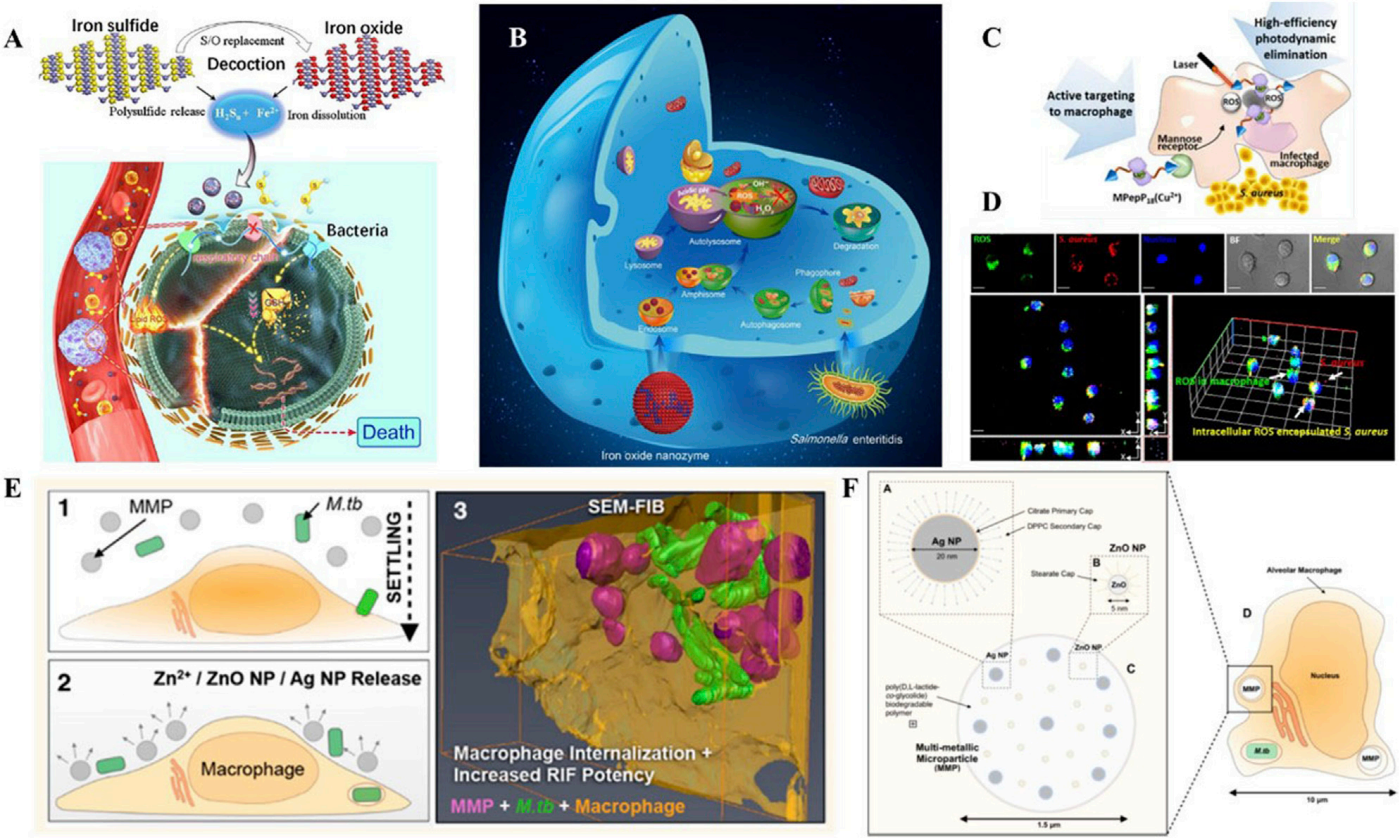
**(A)** Schematic illustration of the bioactive nanoparticles with collaborative bactericidal function depends on both ferrous iron and polysulfide. **(B)** Schematic illustration of the bacteriostatic effects of IONzymes with enzymes-like activity and mechanism responsible for the antibacterial activity. **(C)** Schematic illustration of the peptide-chlorophyll-based photodynamic therapy (PDT) agents to enhance the PDT effect and active targeting property to eliminate intracellular infections. **(D)** Confocal images of intracellular ROS production by MPepP_18_(Cu^2+^) in *S. aureus* infected macrophage. **(E)** Schematic illustration of the biodegradable multi-metallic particles (MMPs), containing Ag NPs and ZnO NPs, and its pulmonary delivery of anti-tuberculous drugs to the endosomal system of *M.tb*-infected macrophages. **(F)** MMPs embedded with Ag NPs and ZnO NPs within the endosome of an *M.tb*-infected alveolar macrophage. Reproduced with permission from Shen et al. (2020); Shi et al. (2018); Cai et al. (2018) (Copyright 2018 American Chemical Society) and Ellis et al. (2018).

Ferrous ions are another type of metal ions that have shown great promise in terms of their antimicrobial biological activity, as they play a critical role in ferroptosis. In a recent study by Shen et al., discovered a multi-nanomaterials combined with ferrous iron and polysulfide (Fe(II)S_n_aq) (Figure 5A) (Shen et al., 2020). The nanomaterials were found to be effective in killing both extracellular and intracellular bacteria, while only having slight toxicity towards host cells. In this system, the sulfur atoms were replaced by oxygen atoms to trigger the release of polysulfides and iron. The collaborative function depended on both ferrous iron and polysulfide. Ferrous irons were able to trigger lipid peroxidation and depress the respiratory chain, which induced ferroptosis-like death within bacteria. At the same time, polysulfide species prevented oxidation of ferrous ions, and demonstrated the ability to oxidize glutathione into GSSG, following with GSH depletion, which leads to DNA degradation and bacteria death.

#### 4.2.2 Nanozymes

The natural process of scavenging pathogens by macrophages involves converting a large amount of hydrogen peroxide in phagosomes into more toxic ROS, catalyzed by enzymes such as oxidase, myeloperoxidase, lipid peroxidase, and other peroxidases. Inspired by this process, there are increased efforts devoted to build artificial enzymes with natural enzyme-like activity to combat extracellular bacteria. Based on their own instinct oxidase enzyme activity, nanozymes can provide various bactericidal ROS, including singlet oxygen, superoxide anion, hypochlorous acid and other oxidation-state components with non- or low-toxic substrates. These excess ROS are capable of damaging bacterial DNA, protein, or nucleic acid, along with breaking cell membranes. Nevertheless, development of nanozymes for efficient production of ROS against intracellular bacteria remains a significant challenge. With these findings in mind, the integration of ROS production and antibiotic delivery may be a promising strategy for efficient elimination both intracellular and extracellular pathogens, especially towards phagosomal bacteria.

Peroxidase is a series of enzymes that catalyze the substrates like hydrogen peroxide into hydroxyl radicals, which are essential in defending pathogens. Presently, enormous nanomaterials have been reported to exhibit the peroxidase-like catalytic activity, including metal, metal sulfide, metal oxide, metal organic frameworks, inorganic materials, and carbon-based materials. Shi et al. designed an iron oxide nanozyme (IONzymes) that can destroy structures, inhibit multiplication, and ultimately cause death of intracellular bacterial (Figure 5B) (Shi et al., 2018). In this work, IONzymes were able to co-localize with intracellular *S. enteritidis* in autophagic vacuoles and regulate ROS levels within acid vacuoles. Moreover, the increasing ROS levels suppressed the survival and reproduction of those pathogens hiding in Leghorn Male Hepatoma-derived cells (LMH).

Oxidases are the major enzymes in peroxisomes, accounting for almost half of the total ones. Generally, the catalytic process of oxidases requires the participation of oxygen, followed by the generation of superoxide anions (Wu et al., 2019). Therefore, nanozymes with oxidase-mimic activity can also be utilized as anti-bacterial materials. Haloperoxidase is another typical category of peroxidase in nature, divided into three subtypes: chloroperoxidase, bromoperoxidase, and iodoperoxidase (ten Brink et al., 2000). Particularly, haloperoxidase can catalyze halide ions in the physiologic environment to hypohalous acid in the presence of acidic and hydrogen peroxide (Hu et al., 2018). Hypohalous acid is a strong oxidizing agent, which can effectively damage bacterial structure (Butler, 1999). Accordingly, haloperoxidase-like nanozymes also have the promising potential for eliminating intracellular bacteria.

Collectively, a vast array of nanozymes present bi-enzymatic and even tri-enzymatic synergism activities, making it preferable to design them as functional components of composite materials.

#### 4.2.3 Photo-active nanomaterials

Photodynamic therapy (PDT) is a method of using photosensitizers remaining in cells to produce singlet oxygen and free radicals to chemically eliminate bacteria. Some biomaterials with PDT effects are used for their active targeting properties to eliminate intracellular infections. Cai et al. (2018) presented a PDT-based strategy to clear *S. aureus* inside macrophages (Figures 5C, D). The dimer coated with peptide−chlorophyll was enabled to active targeting of macrophages, which can generate abundant ROS in infected macrophages with the laser, resulting in high-efficiency photodynamic elimination. This design demonstrated the potential of photodynamic and photothermal effects in intracellular bacterial clearance.

### 4.3 Multifunctional nanomaterials

From above mentioned findings, these bioactive nanomaterials can serve as alternative treatments to traditional antibiotics due to their multiple mechanisms involving intrinsic enzyme-mimic activities, metal toxicity, or physicochemical properties, making them less prone to cause antibiotic resistance. To achieve more efficient synergistic antibacterial effects, researchers are exploring the application of multifunctional nanomaterials integrating the functions of delivery with antibacterial activities (Chang et al., 2017a; Chang et al., 2017b; Lu et al., 2017; Lu et al., 2018; Zhang et al., 2020; Huo et al., 2021; Li et al., 2021; Liu et al., 2021).

Dube et al. presented nanoparticles with chitosan-functionalized shells and PLGA cores for the treatment of tuberculosis (Dube et al., 2014). This rationally designed nanoparticle showed a collaborative function between stimulation of ROS/RNS and delivery of rifampicin. In this case, the combined nanoparticles can not only regulate ROS/RNS levels but also modulate pro-inflammatory cytokine secretion. Meanwhile, it acted as a vehicle to transfer rifampicin inside alveolar macrophages. Similarly, Marwa et al. prepared Ag-coated PLGA particles loaded with pexiganan, which exhibited coordinated antibacterial functions, and were specifically uptaken by macrophages, but not by any non-phagocytic cells (Elnaggar et al., 2020). Additionally, Timothy et al. established composite materials containing Ag and ZnO for the targeted delivery of rifampicin (Figures 5E, F) (Ellis et al., 2018). The sophisticated nanoplatforms were developed to destabilize membranes, increase permeabilization of intracellular *Mycobacterium tuberculosis* and enhance penetration of rifampicin. As a result, multifunctional nanomaterials presented extraordinary bactericidal effects compared to free antibiotics.

## 5 Conclusion and future perspectives

Nanomaterials can enhance preferential accumulation and controlled release of bactericides within pathogens-infected host cells, resulting in increased therapeutic efficiency and reduced potential adverse effects. Size plays a crucial role in the ability of nanomaterial to permeate cells and reach the therapeutic concentration. Positively charged nanomaterials perform excellent interactions with negative-surface bacteria. Moreover, surface-functionalized nanomaterials with special ligands are conducive to an improving targeting of the infected cells. Responsive release at the diseased site can be triggered via pH, enzymes, and redox microenvironment. Beyond providing on-demand delivery of antibiotics, nanomaterials with intrinsic bioactivities, such as metal toxicity, enzyme-like activity and physicochemical properties also show promising potential in combating intracellular pathogens.

However, fabrication of multifunctional nanomaterials for eliminating bacteria is still in infancy, with several fundamental concerns unclear and numerous challenges to be addressed. Positively charged nanomaterials generally present a better cell uptake and adsorption with negative-surface bacteria, but cationic particles tend to cause hemolysis and cytotoxicity. Besides, there are also some examples of negative charged nanomaterials exhibit a higher internalization. Thus, it is worthy to clarify the detailed and wide functions of surface charge in different nanomaterials to balance cell uptake and safety. Despite the effects of size, charge, shape and surface modifications on internalization have been extensively studied, the mechanisms of other properties, such as smoothness and hydrophily, affecting internalization remain indistinct. A comprehensive understanding of the interactions between bacteria, nanomaterials and cellular microenvironment may provide insights into the development of intelligent nanomaterials with precise release property and high level of safety. Besides, nanozyme-based composite materials perform poor selectivity compared with natural enzymes, which might cause undesired toxicity. Additionally, most nanozymes, especially haloperoxidase-mimic ones, are only effective in an acidic microenvironment, limiting their further application. How to compound bioactive and delivering materials to realize synergistic therapeutic effects is highly desired. Lastly, biosafety remains a major concern that determining the translation of nanomaterials against intracellular bacterial infection in clinic.

In summary, we review recent studies on employing multifunctional nanomaterials in treating intracellular infections through targeted delivery of anti-bacterial agents or utilizing their intrinsic bioactivities. We also discuss the opportunities and challenges that need to be focused on in future work. Broadening understanding of the interactions among macrophages, intracellular pathogens and nanoparticles contributes to inspire the development of the next-generation of nanomaterial-based therapeutics against intracellular bacterial infections.
